# Role of Higenamine in Heart Diseases: A Mini-Review

**DOI:** 10.3389/fphar.2021.798495

**Published:** 2022-01-10

**Authors:** Jianxia Wen, Mingjie Li, Wenwen Zhang, Haoyu Wang, Yan Bai, Junjie Hao, Chuan Liu, Ke Deng, Yanling Zhao

**Affiliations:** ^1^ School of Food and Bioengineering, Xihua University, Chengdu, China; ^2^ Department of Pathology, First Affiliated Hospital of Guangxi Medical University, Nanning, China; ^3^ College of Pharmaceutical Science, Yunnan University of Chinese Medicine, Kunming, China; ^4^ Department of Pharmacy, Chinese PLA General Hospital, Beijing, China

**Keywords:** higenamine, heart diseases, pharmacological effects, biological mechanism, mini-review

## Abstract

Higenamine, a natural product with multiple targets in heart diseases, is originally derived from *Aconitum*, which has been traditionally used in China for the treatment of heart disease, including heart failure, arrhythmia, bradycardia, cardiac ischemia/reperfusion injury, cardiac fibrosis, etc. This study is aimed to clarify the role of higenamine in heart diseases. Higenamine has effects on improving energy metabolism of cardiomyocytes, anti-cardiac fibroblast activation, anti-oxidative stress and anti-apoptosis. Accumulating evidence from various studies has shown that higenamine exerts a wide range of cardiovascular pharmacological effects *in vivo* and *in vitro*, including alleviating heart failure, reducing cardiac ischemia/reperfusion injury, attenuating pathological cardiac fibrosis and dysfunction. In addition, several clinical studies have reported that higenamine could continuously increase the heart rate levels of healthy volunteers as well as patients with heart disease, but there are variable effects on systolic blood pressure and diastolic blood pressure. Moreover, the heart protection and therapeutic effects of higenamine on heart disease are related to regulating LKB1/AMPKα/Sirt1, mediating the β2-AR/PI3K/AKT cascade, induction of heme oxygenase-1, suppressing TGF-β1/Smad signaling, and targeting ASK1/MAPK (ERK, P38)/NF-kB signaling pathway. However, the interventional effects of higenamine on heart disease and its underlying mechanisms based on experimental studies have not yet been systematically reviewed. This paper reviewed the potential pharmacological mechanisms of higenamine on the prevention, treatment, and diagnosis of heart disease and clarified its clinical applications. The literature shows that higenamine may have a potent effect on complex heart diseases, and proves the profound medicinal value of higenamine in heart disease.

## Introduction

Natural products represent an important source of compounds used in the discovery of new therapeutic agents (Harvey et al., 2015), which have relatively single compounds with good drug characteristics, including structural diversity and complexity, high selectivity and specific pharmacological activities, as well as novel therapeutic effects and mechanisms of action (Barnett and Stallforth, 2018; Ekiert and Szopa, 2020). The importance of natural products in the prevention and treatment of heart disease is universally known. In the past few decades, a large number of studies have shown that natural products and their related synthetic compounds have good clinical effects on the prevention, treatment and diagnosis of heart disease. Previous studies have revealed that higenamine showed better therapeutic effects on heart disease, including coronary artery disease (CAD), bradyarrhythmia, chronic heart failure (CHF), cardiac ischemia/reperfusion (I/R) injury, cardiac fibrosis, cardiorenal syndrome (CRS), etc., which have aroused great interest of researchers (Chen Z. et al., 2019). In the oriental Asian countries, herbal medicines containing higenamine have been used to treat heart disease for thousands of years. Recently, with extensive studies and clinical reports on higenamine, researchers have proved its beneficial therapeutic effects on various diseases, most prominently on heart disease (Zhang et al., 2017). Based on the effectiveness and safety of clinical medicine practice, higenamine has attracted much attention as a promising chemical compound and natural product for the prevention, diagnosis, and treatment of heart disease.

**FIGURE 1:**
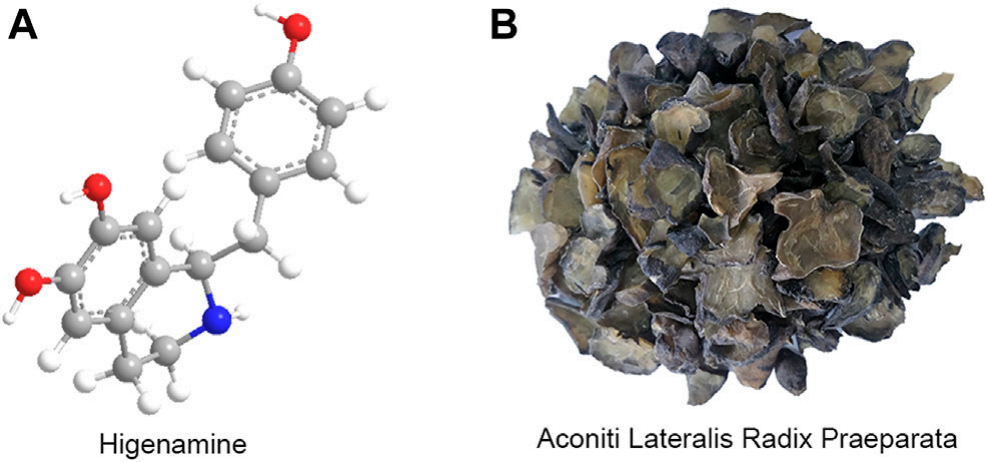
The chemical structure of higenamine **(A)** and the prepared slices of ALRP (Heishunpian) **(B)**.

Higenamine (1-(4-Hydroxybenzyl)-1,2,3,4-tetrahydroisochinolin-6,7-diol), also called as dldemethylcoclaurine or norcoclaurine, is a plant-based benzylisoquinoline alkaloid, which was originally isolated from the root of *Aconitum* (a commonly used traditional Chinese herbal medicine) as an active cardiotonic compound by Kosuge in 1976 (Kosuge and Yokota, 1976). Its molecular formula is C_16_H_17_NO_3_, and its relative molecular mass is 271.311. Its chemical structure is shown in Figure 1A. The 2-(3,4-dimethoxybenzene) acetonitrile could be used as the starting material for the synthesis of higenamine-D4, and heavy water as the stable isotope labeling source (Han et al., 2021). Preliminary studies have found that higenamine has similar pharmacological effects and biological mechanisms to the traditional Chinese medicine (TCM) Aconiti Lateralis Radix Praeparata (ALRP) (Figure 1B), and may be one of the active effect substances of ALRP against CHF. Higenamine also exists in many other medicinal plants, such as Scutellariae barbatae herba, Gnetum montanum Markgr, Asari radix et rhizoma, Nandina domestica, and so on (Kosuge and Yokota, 1976; Li et al., 2020). Besides, higenamine is also commonly used as a dietary supplement, which has a heart-stimulating effect. It is one of the homologous drugs of medicine and food (Calvert et al., 2015; Nuntawong et al., 2020). Structurally, higenamine is similar to catecholamines and can activate α1-, α2-, β1-, and β2-adrenergic receptors (AR). Higenamine is a non-selective β receptor agonist, which has a wide range of effects on heart, brain, vasculature, lung, smooth muscle, striate muscle and so on (Ueki et al., 2011). Higenamine has been identified as a new type of α1-AR antagonist, which contributes to its antihypertensive effect and inhibits platelet aggregation (Zhang et al., 2019). Higenamine acts as a β-AR receptor agonist by acting on dobutamine receptors. Studies have shown that the positive inotropic effect of higenamine and the effect of increasing heart rate (HR) are shorter than that of dobutamine hydrochloride (Shin et al., 1999). Higenamine can play the role of positive chronotropic effect and positive ionotropic effect by regulating β1-AR (Kimura et al., 1994). By regulating the β2-AR, higenamine has the effect of reducing the tension of smooth muscles, thus reflecting the effect of cardiac stimulation (Hudzik et al., 2021). Furthermore, higenamine enhances myocardial contractile response and reduces myocardial cell apoptosis by activating β2-AR (Chen Y. et al., 2019). In the other hand, higenamine also decreases pulmonary inflammation and increases glucose uptake (muscle) (Hudzik et al., 2021). Pharmaceutically, higenamine has multiple pharmacological effects, such as positive inotropic effect, vasodilation, tracheal relaxation, anti-thrombosis, anti-platelet aggregation, anti-inflammatory, anti-apoptosis, anti-oxidative stress, anti-fibrosis, and immune regulation (Pyo et al., 2007; Tsukiyama et al., 2009; Chen et al., 2013; Bai et al., 2019; Guan et al., 2019; Yang et al., 2020). Currently, higenamine has been clinically used for the diagnosis, prevention and treatment of CAD, myocardial perfusion imaging, myocardial I/R injury, bradyarrhythmia, diffuse intravascular coagulation and bronchoconstrictive diseases (Wu et al., 2016; Zhang et al., 2017; Yang et al., 2020).

The chloride salt of higenamine, higenamine hydrochloride [1-(4-Hydroxybenzyl)-1,2,3,4-tetrahydroisoquinoline-6,7-diol hydrochloride], is a white powder, which is more stable than higenamine, and more soluble in water, so it is often used for clinical purposes. Higenamine hydrochloride injection is an original and innovative drug with independent intellectual property rights in China. Higenamine hydrochloride can be used as a cardiac stress test drug for radionuclide myocardial perfusion imaging to assist in the diagnosis and evaluation of myocardial ischemia. It is worth mentioning that higenamine has been approved for clinical research by the China Food and Drug Administration (CFDA). It has been approved by clinical trials to detect coronary artery stenosis and myocardial ischemia by myocardial perfusion imaging with certain specificity and safety (Zheng et al., 2005). Higenamine can be used for radionuclide myocardial perfusion imaging to assist in the diagnosis and assessment of coronary heart disease (CHD) and myocardial ischemia. At present, the phase III clinical trials (2004L02567) of higenamine have been completed and are currently in the evaluation stage of National Medical Products Administration (Zhang et al., 2017). These properties indicate that higenamine has a clear pharmacological effect on the cardiovascular system in clinical practice, but its underlying mechanism is still unclear. Studies specifically designed to evaluate the potential pharmacological effects and biological mechanisms of higenamine in the prevention, diagnosis, and treatment of heart disease are necessary for researchers. Hence, this study provided a synthetic summary of the recent research progress of higenamine on cardiovascular pharmacology and its mechanism of action, thereby contributing to the further clinical practice and application of the drug.

## Methodology

The databases, including PubMed, EMBASE, SinoMed, China National Knowledge Infrastructure (CNKI), VIP medicine information system (VMIS), Wanfang, and Chinese Biomedical Database (CBM), were comprehensive searched. The following search terms were used: “higenamine” [Mesh terms] OR “norcoclaurine” [Mesh terms]. Studies concerning the role of higenamine in heart disease were picked out manually. The related studies were downloaded for further evaluation.

### Ethics Approval and Consent to Participate

Due to this study does not involve animal and patient experiments, the ethics approval and consent to participate are not applicable.

### Pharmacokinetics of Higenamine

Studies have explored the pharmacokinetics of higenamine from humans, rabbits, rats, and dogs in recent years. Feng et al. (2012), gave 10 subjects a continuous intravenous infusion of higenamine, and the dose of higenamine was gradually increased from 0.5 to 4.0 μg kg^−1^ min^−1^, with each administration being 3 min. The pharmacokinetics of higenamine in humans were evaluated by detecting the HR of subjects. The results showed that the peak concentration (C_max_) of higenamine ranges from 15.1 to 44.0 ng mL^−1^. The half-life of higenamine was 0.133 h, and the area under the concentration-time curve (AUC) extrapolated to infinity was 5.39 ng h mL^−1^. The volume of distribution (V) is 48 L. The total clearance (CL) is 249 L h^−1^. Within 8 h, 9.3% of higenamine was recovered in the urine. Studies have shown that higenamine has desirable pharmacokinetic properties. Lo and Chen (1996), administered higenamine to rabbits by intravenous bolus, oral and intravenous infusion, and studied the pharmacokinetics of higenamine in rabbits. The results indicate that AUC increases proportionally with the dose increase of higenamine, and when the dose continues to increase, the percentage of higenamine excreted in the urine remains unchanged. Lo and Chen (1994) found that higenamine is quickly absorbed from the gastrointestinal tract after oral administration. The T_max_ is about 10 min. The cumulative urinary excretion of the same rabbit within 24 h after intravenous (20 mg kg^−1^) and oral administration (50 mg kg^−1^) of higenamine are 4.73 and 0.82%, respectively. Wang et al. (2020), used a non-compartmental model to derive the pharmacokinetic parameters of higenamine in the plasma of rats. Oral administration of higenamine in the dose of 3.0–30.0 mg kg^−1^ could quickly reach its maximum concentration, and the T_max_ for all doses was 0.42 h. The higher the total level of the athlete’s intake of food, the longer it takes for the metabolism to eliminate higenamine. Zheng et al. (2004), gave intravenous injection of higenamine to beagle dog. This study found that the dog’s metabolism under this condition conforms to the two-compartment model, with t_1/2_ of 8.60 min, indicating that the pharmacokinetics of higenamine in different species of animals may be different. However, animal experiments have certain limitations, and further research on health and pharmacokinetics in patients is needed. The pharmacokinetics parameter of higenamine in different kinds of animals is shown in Table 1.

**TABLE 1:** Pharmacokinetics parameters of higenamine in different kinds of animals.

Species	Dose of higenamine	Pharmacokinetics parameter	Findings
Human Feng et al. (2012)	22.5 μg kg^−1^, *i.v.*	AUC_last_ 5.31 ± 1.21 ng h mL^−1^	Two-compartment pharmacokinetic model
		AUC_0-inf_ 5.39 ± 1.23 ng h mL^−1^	
		C_max_ 31.3 ± 9.24 μg L^−1^	
		CL 249 ± 42.78 L h^−1^	
		CL_r_ 22.9 ± 4.41 L h^−1^ t_1/2_ 0.133 ± 0.02 h	
		V 48 ± 13.83 L	
		A_e_ 120.6 ± 34.5 μg fe% 9.3 ± 2.2%	
Rabbits Lo and Chen (1996)	NR	t_1/2_ 22 min	Two-compartment open pharmacokinetic model
		total body clearance 127.7 ml min^−1^ kg^−1^	
		mean residence time 9.28 min	
		volume of distribution at steady state 1.44 L kg^−1^	
		fraction of urinary excretion 5.48%	
Rabbits Lo and Chen (1994)	50 mg kg^−1^, *p.o.*	T_max_ 10 min Cumulative urinary excretion 0.82%	Two-compartment model
	20 mg kg^−1^, intravenous	Cumulative urinary excretion 4.73%	
Rats Wang et al. (2020)	30.0 mg kg^−1^, *i.g.*	AUC_(0-t)_ 11,482.55 ± 1,291.49 ng mL^−1^ min^−1^	High concentrations of topical or oral use of higenamine-rich materials may cause positive test of higenamine in the urine of athletes
		AUC_(0-∞)_ 13,030.94 ± 714.93 ng mL^−1^ min^−1^ t_1/2z_ 53.67 ± 26.11 min	
		T_max_ 25.83 ± 2.04 min	
		C_max_ 256.38 ± 37.33 Ng mL^−1^	
		MRT_(0-t)_ 53.02 ± 1.66 min	
		MRT_(0-∞)_ 76.16 ± 19.93 min	
		VRT_(0-t)_ 1,146.09 ± 89.10 min^2	
		VRT_(0-∞)_ 3,183.12 ± 763.67 min^2	
		Vz/F 180.47 ± 93.67 L kg^−1^	
		CLz/F 2.31 ± 0.12 L min^−1^ kg^−1^	
	15.0 mg kg^−1^, *i.g.*	AUC_(0-t)_ 3,824.53 ± 332.08 ng mL^−1^ min^−1^	
		AUC_(0-∞)_ 4,051.78 ± 280.95 ng mL^−1^ min^−1^ t_1/2z_ 36.00 ± 9.10 min	
		T_max_ 25.00 ± 0.00 min	
		C_max_ 119.00 ± 23.05 ng mL^−1^	
		MRT_(0-t)_ 47.77 ± 1.75 min	
		MRT_(0-∞)_ 55.76 ± 5.66 min	
		VRT_(0-t)_ 1,000.72 ± 76.25 min^2	
		VRT_(0-∞)_ 2,354.15 ± 878.73 min^2	
		CLz/F 3.72 ± 0.26 L min^−1^ kg^−1^	
		Vz/F 194.31 ± 54.11 L kg^−1^	
	3.0 mg kg^−1^, *i.g.*	AUC_(0-t)_ 801.78 ± 65.96 ng mL^−1^ min^−1^	
		AUC_(0-∞)_ 849.59 ± 76.10 ng mL^−1^ min^−1^ t_1/2z_ 34.69 ± 12.13 min	
		T_max_ 25.00 ± 0.00 min	
		C_max_ 24.46 ± 3.44 ng mL^−1^	
		MRT_(0-t)_ 44.75 ± 1.98 min	
		MRT_(0-∞)_ 50.89 ± 2.57 min	
		VRT_(0-t)_ 890.10 ± 104.91 min^2	
		VRT_(0-∞)_ 1944.89 ± 551.80 min^2	
		CLz/F 3.55 ± 0.32 L min^−1^ kg^−1^	
		Vz/F 174.90 ± 49.02 L kg^−1^	
	3.0 mg kg^−1^, *i.v.*	AUC_(0-t)_ 25,966.04 ± 759.68 μg L^−1^ min^−1^	
		AUC_(0-∞)_ 26,185.37 ± 787.28 μg L^−1^ min^−1^ t_1/2z_ 26.92 ± 3.31 min	
		T_max_ 2.00 ± 0.00 min	
		C_max_ 2,762.33 ± 113.72 μg L^−1^	
		MRT_(0-t)_ 11.21 ± 0.25 min	
		MRT_(0-∞)_ 12.37 ± 0.29 min	
		VRT_(0-t)_ 283.75 ± 13.36 min^2	
		VRT_(0-∞)_ 464.88 ± 34.75 min^2	
		Vz/F 4.45 ± 0.55 L kg^−1^	
		CLz/F 0.11 ± 0.003 L min^−1^ kg^−1^	
Dog Zheng et al. (2004)	10 mg kg^−1^, single *i.v.*	AUC_(0–30)_ 0.076 ± 0.00027 mg min L^−1^ t_1/2β_ 8.60 ± 0.26 min	Two-compartment pharmacokinetic model
		CL_T_ 0.13 ± 0.0058 L min^−1^ kg^−1^	
		V/F 0.95 ± 0.0038 L kg^−1^	

Notes: NR, not report; AUC, area under the concentration-time curve; CL, total clearance.

## Effects of Higenamine on Heart Disease In Vivo

### Anti-Heart Failure

Heart failure (HF) is myocardial damage caused by any cause such as myocardial infarction (MI), cardiomyopathy, hemodynamic overload, inflammation, etc., resulting in changes in myocardial structure and function, and finally resulting in hypofunction of ventricular pumping or filling. The main clinical manifestations are dyspnea, fatigue and fluid retention. CHF is the terminal stage of various heart disease, which refers to a state of persistent HF that can be stabilized, worsened, or decompensated (Wen et al., 2020b). Due to high morbidity and high mortality, CHF is still a clinical cause that seriously endangers the health of patients with various heart disease (Dini et al., 2018). In recent years, the relationship between CHF and myocardial energy metabolism has become a hot spot in clinical research. Even though the drugs used to treat CHF are diverse, the drugs themselves can enhance the energy metabolism of myocardial mitochondria and may also have the preventing and therapeutic effects on CHF (Wen et al., 2019a) (Table 2).

**TABLE 2:** *In vivo* pharmacological activities of higenamine in heart disease.

Effects	Animals	Experimental model	Doses of higenamine	Pathways
CHF Wen et al. (2020b), Wen et al. (2020c)	Rats	DOX (15 mg kg^−1^, *i.p.*)-induced CHF	5 mg kg^−1^	Regulating LKB1/AMPKα/Sirt1 signa ling pathway
Anti-I/R injury Wu et al. (2016)	Mice	I/R-induced MI	10 mg kg^−1^	Mediating the β2-AR/PI3K/AKT cascade
Myocardial damages Lee et al. (2006)	Rats	Myocardial I/R injury	1–10 mg kg^−1^ (*i.p.*)	Induction of heme oxygenase-1
Cardiac fibrosis Zhu et al. (2021)	Mice	TAC or ISO (50 mg kg^−1^ *i.p.*)-induced cardiac fibrosis	10 mg kg^−1^	Suppressing TGF-β1/Smad signaling
CRS Deng et al. (2020)	Rats	Left anterior descending coronary artery ligation combined with 5/6 STNx	0.5–4.5 mg kg^−1^	Targeting ASK1/MAPK (ERK, P38)/NF-kB signaling pathway

Notes: CHF, chronic heart failure. I/R, ischemia/reperfusion. CRS, cardiorenal syndrome; DOX, doxorubicin; ISO, isoproterenol; TAC, transverse aortic constriction; STNx, subtotal nephrectomy. *i.p*, intraperitoneal injection.

A number of studies by our team showed that higenamine exerts a therapeutic effect on doxorubicin (DOX)-induced CHF *via* the cardiotonic effect and promoting myocardial energy metabolism. Higenamine had effects on ameliorating heart function, down-regulation serum indices, alleviating histological damage of heart tissue and reducing the apoptosis of myocardial cells (Wen et al., 2020b; Wen et al., 2020c). Specifically, higenamine increased the haemodynamic parameter levels of left ventricular systolic pressure (LVSP) and maximum rate of increase in left ventricular pressure (+dp/dt_max_), but decreased left ventricular end-diastolic pressure (LVEDP) and maximum rate of decrease in left ventricular pressure (−dp/dt_max_). Moreover, higenamine decreases serum level of neuro-humoral factor, such as renin, angiotension II (Ang-II), aldosterone (ALD), and endothelin-1 (ET-1); serum level of myocardial biomarkers, such as brain natriuretic peptide (BNP), NT-proBNP, lactate dehydrogenase (LDH), creatine kinase-MB (CK-MB), and aspartate aminotransferase (AST); but increases the serum level of adenosine phosphate, such as adenosine triphosphate (ATP), ATPase, nicotinamide adenine dinucleotide (NAD), and NADH in CHF rats induced by DOX (Wen et al., 2020b; Wen et al., 2020c). Serum metabolomics analyses indicated that the therapeutic effects of higenamine on CHF rats were primarily related to the comprehensively regulation of mitochondrial energy metabolism metabolites, including acetylphosphate, coenzyme A, 3-Carboxy-1-hydroxypropylthiamine diphosphate, PE (O-18:1 (1Z)/20:4 (5Z, 8Z, 11Z, 14Z)), lysoPC(18:1 (9Z)), oleic acid, palmitic acid, and PC(16:0/16:0). Pathway analysis showed that higenamine on CHF treatment was related to energy metabolism signaling pathways, including glycerophospholipid metabolism, linoleic acid metabolism, fatty acid metabolism, citrate cycle (TCA cycle), arachidonic acid metabolism, pantothenate and CoA biosynthesis, and pyruvate metabolism (Wen et al., 2020b). The potential mechanism of these activities was partially related to down-regulating renin-angiotensin-aldosterone system (RAAS) pathway-related molecules and up-regulating LKB1/AMPKα/Sirt1-related pathway (Wen et al., 2020c) (Table 2). Moreover, studies focused on ALRP, one of the sources of higenamine, was in accordance with these results showing the alleviating of mitochondrial energy metabolism. ALRP could significantly improve the left ventricular function and cardiac enzyme activities *via* activating the PPARα/PGC-1α/Sirt3 signaling pathway, which promotes mitochondrial energy metabolism and protects against CHF (Wen et al., 2019b) (Table 2). Another study further indicated that ALRP could regulate the metabolites related to the mitochondrial energy metabolism pathway, which increases the relative gene expression level of energy metabolism, including *PPARδ*, *PPARγ*, *Lpl*, *Scd*, *Fasn*, and *Pla2g2e* (Wen J. X. et al., 2020). These findings all indicate that higenamine plays a role in the treatment of CHF by regulating the mitochondria energy metabolism of myocardial.

### Reducing Cardiac Ischemia/Reperfusion Injury

Cardiac I/R injury after myocardial ischemia is a partial or complete acute obstruction of the coronary artery and recanalization after a certain period of time. Although the ischemic myocardium can be restored to normal perfusion, the tissue damage is a progressive pathological process (Tibaut et al., 2017). A series of damaging changes such as myocardial ultrastructure, energy metabolism, cardiac function and electrophysiology caused by ischemic period will be more prominent after vascular recanalization, and severe arrhythmia may even occur and cause sudden death. In the process of I/R, myocardial cell apoptosis will aggravate the development of ischemic heart injury and HF. I/R injury significantly increases morbidity and mortality after MI (Herr et al., 2020). Wu et al. (2016), reported that higenamine reduces I/R-induced MI in mice *in vivo*. In addition, higenamine stimulates AKT phosphorylation and activates PI3K to play an anti-apoptotic effect in cardiomyocytes. These results indicated that the heart protection effects of higenamine are mediated by the β2-AR/PI3K/AKT cascade (Table 2). Lee et al. (2006), found that administration of higenamine (bolus injection, intraperitoneal injection) 1 h before I/R injury dramatically reduced the release of cytochrome c, caspase-3 activity and Bax expression in the left ventricle of rats, but increased the expression of heme oxygenase-1 (HO-1). I/R-induced myocardial injury is related to mitochondrial-dependent apoptosis. This study also confirmed the key role of HO-1 in the protective effect of higenamine in the myocardial injury induced by I/R (Table 2). Therefore, higenamine is expected to become a potential agent for the treatment of myocardial ischemic injury.

### Attenuating Pathological Cardiac Fibrosis and Dysfunction

Cardiac fibrosis is the result of persistent and/or repeated myocardial ischemia and hypoxia caused by moderate to severe coronary atherosclerotic stenosis, leading to the gradual development of chronic ischemic heart disease (IHD). Myocardial fibrosis is the inevitable process of the development of various clinical heart disease to the final stage, and is the main manifestation of cardiac structural remodeling. It is currently believed to be closely related to arrhythmia, cardiac dysfunction and even sudden cardiac death. It is mainly characterized by the proliferation of fibroblasts and the deposition of extracellular matrix (ECM). The deposition of ECM causes an increase in the stiffness of the heart and a decrease in compliance, which affects the normal diastolic and contractile functions of the heart (Frey and Olson, 2003). It is currently believed that the pathogenesis of cardiac fibrosis involves multiple influencing factors, including the endothelial-to-mesenchymal transition (EndMT), left ventricle pressure overload, inflammation activation, the effector cells and cellular mediators of immune system in cardiac tissue (Burke et al., 2019; Martinez-Martinez et al., 2019; Liu et al., 2020; Wilhelmi et al., 2020).

In the transverse aortic constriction (TAC) and isoproterenol (ISO) injection-induced cardiac fibrosis model, higenamine in the dose of 10 mg kg^−1^ in mice could abolish the decreased fractional shortening (FS) and ejection fraction (EF) as well as increased systolic left ventricular internal diameter (LVIDs) and systolic left ventricular volumes (LVVs) in cardiac fibrosis mice induced by TAC and ISO. Moreover, the extremely increased heart size, ratio of heart weight to tibia length (HW/TL), and ratio of heart weight to body weight (HW/BW) induced by TAC and ISO was also decreased by higenamine treatment. In addition, higenamine exerts a therapeutic effect on TAC-induced cardiac dysfunction, as well as ISO-induced cardiac remodeling and fibrosis in mice by attenuating serum level of hypertrophic markers, such as β-myosin heavy chain (β-MHC), A-type natriuretic peptide (ANP), and BNP. These results indicated that HG remarkably inhibits myocardial fibrosis in different models by inhibiting cardiac fibrosis activation. The potential mechanism of higenamine exerting these pharmacological activities is related to the inhibition of TGF-β1/Smad signaling pathway (Zhu et al., 2021) (Table 2).

### Ameliorating Cardiac and Renal Fibrosis

Cardiorenal syndrome (CRS) is a clinical syndrome characterized by the co-pathogenicity of the heart and kidneys. It refers to the acute or chronic dysfunction of one organ resulting in progressive dysfunction of the other organ, and ultimately caused to the failure of both organs (Rangaswami et al., 2019). Currently, higenamine is considered to be effective in the treatment of CRS. A study from Deng (Deng et al., 2020) revealed that compared with type 2 CRS rats, rats treated with higenamine (0.5–4.5 mg kg^−1^) showed significantly increased LV ejection fraction (LVEF%) and left ventricular fraction shortening (LVFS%), decreased LV end systolic volume (LVESV), LV posterior wall thickness LVPW, Cardiac weight index (CWI) and kidney weight index (KWI). In addition, higenamine markedly decreased serum creatinine (Scr), blood urea nitrogen (BUN), indole sulfate (IS), and 24-h urine protein level as well as memorably diminished cardiac and renal fibrosis in CRS rats, accompanied with the reduced protein level of α-smooth muscle actin (α-SMA), transforming growth factor-β1 (TGF-β1), and collagen I. Moreover, it dramatically improved left ventricular remodeling and systolic function in CRS. This heart and renal therapeutic effects are strongly relevant to directly inhibited the protein expression of phosphorylated apoptosis signal-regulated kinase 1 (p-ASK1) and its downstream mitogen-activated protein kinases (MAPK) (ERK, P38)/NF-kB in cardiorenal tissues of CRS rats. This study finally found that higenamine alleviated ventricular remodeling and renal fibrosis *via* targeting ASK1/MAPK (ERK, P38)/NF-kB signaling pathway (Table 2).

## Effects of Higenamine on Heart Disease In Vitro

Most studies of higenamine in the treatment of heart disease *in vitro* are based on its role as α and β receptor agonists. Higenamine could increase the contractility, contraction frequency and contraction amplitude of guinea pig ventricular papillary muscle, mouse left atrium and mouse right atrium in a concentration-dependent range of 0.1–800 μM, and increase the left atrial tension of rabbit. However, these effects were competitively blocked by the non-selective β-AR blocker propranolol, indicating that higenamine exerted a cardiotonic effect by stimulating β-AR (Park et al., 1984; Kimura et al., 1989; Kimura et al., 1994; Kimura et al., 1996). Higenamine in the concentration of 10 and 100 μM could increase the contraction frequency and contraction amplitude of primary cultured neonatal rat ventricular myocytes (Han et al., 1981). Chen et al. (2019b), investigated the contractility of higenamine and ISO on adult rat cardiomyocytes. Studies have found that the stimulation of higenamine on cardiomyocytes was dose-dependent. The EC_50_ of its contractile response was 0.33 μM, which was equivalent to the EC_50_ measured by cAMP of 0.129 μM, but it was not as effective as ISO. The β1-or β2-receptor antagonists completely blocked the positive inotropic effect of higenamine on cardiomyocytes. Wang et al. (2019) found that higenamine controled the electrophysiology of the heart by having a dominant effect on the sinus node without inducing ectopic activities that cause arrhythmia, which might help treat bradycardia. Higenamine could inhibit the apoptosis of ventricular myocytes in primary neonatal rats and adult mice, and its anti-apoptotic effect could be completely eliminated by β2-AR, but could not be antagonized by β1-AR. Higenamine reduces myocardial damage caused by ischemia/reperfusion in a β2-AR-dependent manner, thereby exerting anti-apoptosis and protecting the heart (Wu et al., 2016) (Table 3). Studies have also indicated that higenamine at a concentration of more than 30 μM could inhibit the proliferation and migration of rat aortic smooth muscle cells in a concentration-dependent manner. It was speculated that higenamine might have the effect of preventing the restenosis of blood vessels in various heart disease, such as atherosclerosis, allograft vascular disease, hypertension, and angioplasty (Weng and Sun, 2020). Zhang et al. (2019), indicated that higenamine can inhibit the production of inositol monophosphate. It also inhibits the influx and entry of calcium ions induced by phenylephrine (PE) and the phosphorylation of extracellular signal-regulated kinases 1 and 2. In addition, higenamine has similar affinities (pKi) to the cloned α1A-, α1B- and α1D-AR, which may contribute to its antihypertensive effect (Table 3).

**TABLE 3:** *In vitro* pharmacological activities of higenamine in heart disease.

Effects	Cells/tissues	Experimental model	Concentrations of higenamine	Targets/pathways
Treatment of blood pressure Zhang et al. (2019)	HEK293 cell lines	Stably transfected with α1A-, α1B-, and α1D-AR/Flag and treatment with 10 μM PE	10 μM	Antagonist for α1-AR
Improve energy metabolism of cardiomyocytes Wen et al. (2019a), Wen et al. (2020c)	H9c2 cells	5 μM DOX-induced H9c2 cells injury	5–20 μM	Upregulation the PPARα/PGC-1α/Sirt3 pathway
Treatment of CHF Chen et al. (2019a)	SK/SK-β2 cells adult rat cardiomyocytes cells	0.001 μg mL^−1^ PTX	0.1 μM	Stimulating the Gs and Gi pathways in β2-AR signaling
		Perfused with Ca^2+^-free perfusion buffer		
Anti-cardiac fibroblast activation Zhu et al. (2021)	AMCM	10 μM PE-induce hypertrophic growth of AMCM	100 μM	Inhibiting TGF-β1/Smad signaling
	NRCF	NRCF cells were stimulated with 10 ng mL^−1^ TGF-β1		
Anti-cardiorenal fibrosis Deng et al. (2020)	NRCM	10 μM IS	0.01–100 μM	Mediateing ASK1 and its downstream MAPK (pERK, p-P38) and p-NF-kB pathways
	NRCF	10 ng mL^−1^ TGF-β1		
Anti-oxidative stress and apoptosis in cardiomyocytes Chen et al. (2013)	NRCM/H9c2 cells	5 μM DOX-induced cardiotoxicity	0.5–50 μM	Activating the PI3K/Akt signaling pathway
Anti-myocyte apoptosis Wu et al. (2016)	NRCM/AMVM	H_2_O_2_ (250 μM for 24 h) in NRVM	100 μM in NRVMs/100 μM in AMVMs	β2-AR/PI3K/AKT signaling pathway
		H_2_O_2_ (20 μM for 24 h) in AMVM		

Notes: CHF, chronic heart failure; AMCM, adult mouse cardiac myocytes; AMVM, adult mouse ventricular myocytes; NRCF, neonatal rat cardiac fibroblasts; NRCM, neonatal rat cardiac myocyte; NRVM, neonatal rat ventricular myocytes. SK, SK-N-MC, cell.

### Improve Energy Metabolism of Cardiomyocytes

The heart is an energy-intensive organ that consumes large amounts of ATP every day to provide fuel for pumping functions (Jiang et al., 1980; Neubauer, 2007). Since mitochondria are organelles that coordinate multiple metabolic systems and enzymes involved in substrate utilization and oxidative phosphorylation, mitochondrial metabolic dysfunction plays a key role in energy metabolism. Therefore, drugs that enhance myocardial mitochondrial energy metabolism and respiratory function of cardiomyocytes may have the potential effects on treating heart disease (Doenst et al., 2013). In our previous studies, 5 μM DOX was used to establish a cardiomyocyte injury model to simulate myocardial cell energy metabolism disorder and respiratory injury during CHF *in vitro*. Cell metabolomics analyses indicated that the protective effects of higenamine on DOX-induced mitochondrial energy metabolism disorder and respiratory dysfunction were closely associated with the mitochondrial energy metabolism metabolites, including pantothenic acid, palmitic acid, eicosanoyl-CoA, coenzyme A, 1,4-beta-D-Glucan, oleic acid, and so on. Metabolic pathway analysis indicated that these potential metabolites were related to energy metabolism signaling pathways, including citrate cycle (TCA cycle), biosynthesis of unsaturated fatty acids, fatty acid metabolism, fatty acid biosynthesis, pentose and glucuronate interconversions, and so on (Wen et al., 2020c). Besides, higenamine could meliorate DOX-induced mitochondrial dysfunction and elevate cell mitochondrial oxygen consumption rate (OCR) as well as extracellular acidification rate (ECAR), thus enhancing the mitochondrial function of H9c2 cardiomyocytes (Wen et al., 2019a; Wen et al., 2020c) (Table 3). Molecular biological mechanism research suggests that the protective mechanism of higenamine on ameliorating DOX-induced mitochondrial function impairment in H9c2 cells may be related to the upregulation of the PPARα/PGC-1α/Sirt3 pathway, which promotes mitochondrial energy metabolism and protects against heart disease (Wen et al., 2019a) (Table 3). In addition. Chen Y. et al. (2019), applied contractility experiments to prove that higenamine exerts a positive inotropic effect by stimulating β1/β2-AR, and has no preference for stimulating the Gs and Gi pathways in β2-AR signaling. The pharmacological effects of higenamine in the treatment of CHF and the mechanism of its cardiotoxicity have been elucidated.

### Anti-Cardiac Fibroblast Activation

Cardiomyocyte hypertrophy is crucial in pathological heart remodeling as well as HF. Zhu et al. (Liu et al., 2020), investigated the effect of higenamine on cardiomyocytes hypertrophy in adult mouse cardiac myocytes (AMCM) *in vitro*. Studies have found that higenamine has no significant effects on the cardiomyocytes hypertrophy induced by PE, but it could dose-dependently inhibit the relative mRNA and protein expression of α-SMA in TGF-β1 stimulated neonatal rat cardiac fibroblasts (NRCF). In addition, higenamine could reduce the relative mRNA expressions of collagen I, collagen III and fibronectin in the TGF-β1 stimulated NRCF. These results indicated that higenamine improves cardiomyocyte fibrosis and dysfunction by inhibiting TGF-β1/Smad signaling and cardiac fibroblasts activation. Deng et al. (2020), also found that higenamine dramatically inhibits the collagen synthesis of NRCF and inhibits the hypertrophy of neonatal rat cardiomyocytes. It mainly mediates ASK1 pathways to relieve cell fibrosis (Table 3).

### Anti-Oxidative Stress and Apoptosis

Recently, studies have shown that higenamine could protect cardiomyocytes through its anti-oxidative stress injury and anti-apoptosis effects (Chen et al., 2013; Wu et al., 2016). Chen et al. (2013) (Table 3), studied the cardioprotective mechanism of higenamine in DOX-induced cytotoxic neonatal rat cardiac myocyte (NRCM) and H9c2 cells *in vitro*. The results showed that higenamine could increase the cell viability of DOX-injured cardiomyocytes, increase SOD activity, reduce the generation of ROS and the formation of MDA, and inhibit the release of LDH and the inherent mitochondrial-dependent apoptosis pathway of cardiomyocytes. Molecular biology studies indicated that higenamine played a cardioprotective effect on DOX-induced cardiotoxicity by activating the PI3K/Akt signaling pathway. Wu et al. (2016), reported that higenamine inhibits apoptosis of primary neonatal rat cells and adult mouse ventricular myocytes, and reduces the levels of cleaved caspase- 3 and 9 as a biochemical marker of apoptosis *in vitro*. Higenamine stimulates AKT phosphorylation and requires PI3K activation to exert anti-apoptotic effects in cardiomyocytes. The anti-apoptotic effect of higenamine is mediated by the β2-AR/PI3K/AKT cascade (Table 3).

## Effects of Higenamine on Heart Disease in Clinical

Higenamine has been clinically studied in China, which can be used as a pharmacological agent for cardiac stress test and for the treatment of a variety of heart disease. The subjects of the clinical study included healthy volunteers, sick sinus syndrome, heart block, hypertension and other heart disease patients (Feng et al., 2012; Jiang et al., 1980; Jiang et al., 1981a; Jiang et al., 1981b; Bao et al., 1982; Liu et al., 1983; Du et al., 2007; Cao et al., 2012a; Du et al., 2014). Clinical studies on higenamine intervention in heart disease are listed in Table 4. The number of subjects included in the study ranged from 10 to 120 (Jiang et al., 1980; Jiang et al., 1981a; Jiang et al., 1981b; Bao et al., 1982; Liu et al., 1983; Du et al., 2007; Cao et al., 2012a; Feng et al., 2012; Du et al., 2014). The research objectives include pharmacokinetic and pharmacodynamics study, tolerability study, pharmacological stress agent, suitability as pharmacological stress agent, effect on the function of left ventricle, effects on sick sinus syndrome, effect on patients with heart block, effect on patients with heart bloc and so on. The intervention of higenamine include intravenous infusions escalating from 0.5 to 4 mg kg^−1^ min^−1^ (Du et al., 2007; Cao et al., 2012a; Feng et al., 2012; Du et al., 2014), 2.5 or 5 mg higenamine intravenous slow infusion (Jiang et al., 1980a; Jiang et al., 1980b; Jiang et al., 1981; Bao et al., 1982; Liu et al., 1983). The results of the study found that higenamine could continuously increase the HR levels of subjects, but there are variable effects on systolic blood pressure (SBP). diastolic blood pressure (DBP). Higenamine has increasing effects on cardiac output, and several studies have not reported it (Table 4).

**TABLE 4:** Clinical research of higenamine in heart disease.

Subjects	Cases	Effects	Intervention	HR	SBP	DBP	Cardiac output
Healthy volunteers Feng et al. (2012)	10	Pharmacokinetic and pharmacodynamics study	Higenamine intravenous infusions escalating from 0.5 to 4 mg kg^−1^ min^−1^ for 3 min	↑	-	↓	NR
Healthy volunteers Du et al. (2007)	32	Tolerability study	Higenamine intravenous infusions escalating from 0.5 to 4 mg kg^−1^ min^−1^	↑	-	↓	NR
Confirmed or suspected heart disease Du et al. (2014)	120	Pharmacological stress agent	Higenamine intravenous infusions escalating from 0.5 to 4 mg kg^−1^ min^−1^	↑	-	↓	NR
Suspected heart disease Cao et al. (2012a)	71	Suitability as pharmacological stress agent	Higenamine intravenous infusions escalating from 0.5 to 4 mg kg^−1^ min^−1^	↑	-	↓	NR
heart disease Liu et al. (1983)	15	Tolerability study	2.5 mg Higenamine intravenous slow infusion	↑	NR	NR	↑
heart disease Jiang et al. (1981b)	19	effect on the function of left ventricle	2.5 mg higenamine intravenous slow infusion	↑	↑	↓	↑
Sick sinus syndrome Jiang et al. (1981a)	22	Effects on sick sinus syndrome	2.5 mg higenamine intravenous slow infusion	↑	NR	NR	NR
Heart block Bao et al. (1982)	14	effect on patients with heart block	5 mg higenamine intravenous slow infusion	↑	↑↓	↑↓	NR
Heart block Jiang et al. (1980)	68	effect on patients with heart bloc	2.5 mg higenamine intravenous slow infusion	↑	↑↓	↑↓	NR

Notes: HR, heart rate; SBP, systolic blood pressure; DBP, diastolic blood pressure. -, no change. NR, not report. Min, minute(s). ↑, increased. ↓, decreased. ↑↓, variable effects.

### Safety of Higenamine in the Treatment of Heart Disease

The toxic effects of higenamine mainly affect the cardiovascular and nervous systems (Chan, 2009; Chen et al., 2012). Cardiovascular features include high blood pressure, chest pain, palpitations, and bradycardia. Studies have shown that in isolated mouse atria, higenamine can enhance myocardial contractility and aconitine-induced tachyarrhythmia (Kimura et al., 1994), and increase the pulsation of cultured cardiomyocytes. Higenamine produces positive inotropic effects by acting on cardiac AR, which can enhance myocardial contractile response and reduce myocardial cell apoptosis to play a pharmacological role in the treatment of CHF (Park et al., 1984). However, higenamine caused the activation of β1-AR to induce CAMKII-dependent cell death and cardiac remodeling. It shows that the toxicity of aconitum is at least partly caused by aconitine.

The median lethal dose (LD_50_) of higenamine in intravenous injection, intraperitoneal injection and oral administration are 58.9 mg kg^−1^, 300 mg kg^−1^, and 3.35 g kg^−1^ in mice, respectively. Regarding cardiovascular regulation, higenamine can significantly increase blood pressure, enhance myocardial contractility, speed up HR and expand coronary blood vessels. However, the effect of higenamine in accelerating the HR and increasing myocardial oxygen consumption limits its clinical use. On the other hand, the rapid and controllable features of higenamine can be used to diagnose myocardial ischemia clinically. A study reported that 48 human subjects in the higenamine group took it daily for 8 weeks. This study found that a daily higenamine supplement for 8 weeks alone or in combination with caffeine and yohimbe bark extract do not cause substantially changes in resting HR, breathing rate, blood pressure, and liver enzyme activity of men. It shows the safety of higenamine to human subjects. It is worth noting that the saturation pharmacodynamic model fully describes the increase in the HR of healthy Chinese volunteers after giving higenamine. Zhang et al. (2002), found that the hemodynamic effects of higenamine and dobutamine on dogs were similar, and they were still tolerable even at a dose of 500 μg kg^−1^ min^−1^, and there were no serious adverse reactions. It had good safety and could be used as a substitute for dobutamine. Continuously taking higenamine capsules (50 mg per capsule) for 8 weeks, it had no significant effect on men’s resting breathing rate, HR, blood pressure, urine test indicators, complete blood count, metabolic indicators, liver enzyme activity, and blood lipids, indicating that oral higenamine has a certain degree of safety (Bloomer et al., 2015). Clinical studies have found that patients receiving higenamine treatment have been reported to have varying degre of dyspnea, palpitations, dizziness, headache, chest tightness and other adverse reactions (Cao et al., 2012b; Zhou et al., 2012). The toxic effects of higenamine mainly affect the cardiovascular and nervous systems (Chan, 2009; Chen et al., 2012). Cardiovascular features include high blood pressure, chest pain, palpitations, and bradycardia (Du et al., 2014). Higenamine can enhance myocardial contractility and aconitine-induced tachyarrhythmia, and increase the pulsation of cultured cardiomyocytes in isolated mouse atria (Kimura et al., 1994). Thus, it should be comprehensively evaluated while using higenamine for preventing, treating and diagnosing heart disease according to the actual situation during clinical use.

## Conclusion and Perspective

Higenamine hydrochloride injection is an original innovative drug with independent intellectual property rights in China, and it can be used as a diagnostic drug for cardiac stress test. Currently, higenamine has obtained the implicit permission of clinical trials. It has certain specificity and safety in the detection of coronary artery stenosis and myocardial ischemia by myocardial perfusion imaging (Zheng et al., 2005). It can be used for radionuclide myocardial perfusion imaging to assist diagnosis and evaluation of CHD and myocardial ischemia, and has completed the phase III clinical study (2004L02567), and is in the review stage of the National Medical Products Administration (Zhang et al., 2017).

**FIGURE 2:**
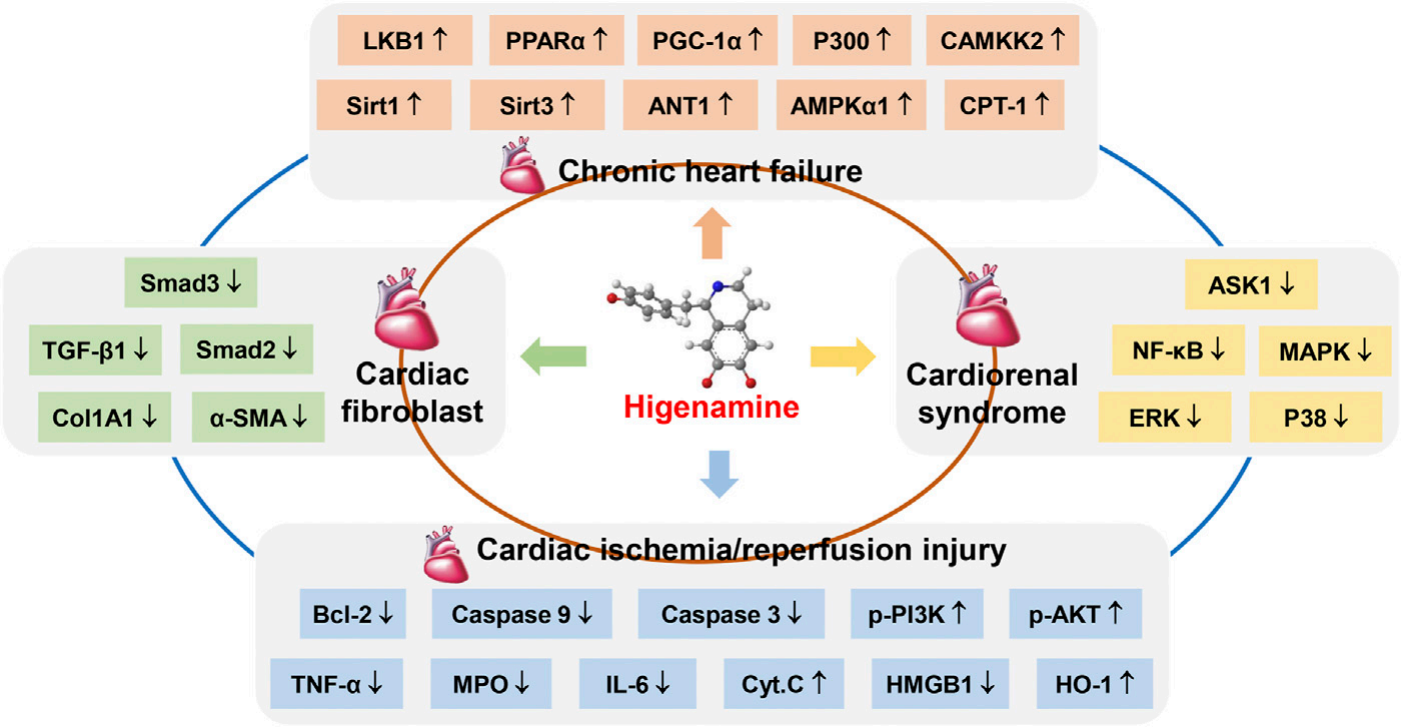
The pharmacological effect of higenamine on heart disease through multiple targets. ↑, Higenamine has an agonistic effect on this target. ↓, Higenamine has an inhibitory effect on this target.

This study reviewed the pharmacological effects and biological mechanisms of higenamine intervention in heart disease. Taken together, higenamine has a significant alleviate effect on heart disease such as CHF, cardiac fibroblast, CRS, and cardiac I/R injury, which could significantly increase the relative mRNA and protein expression levels of LKB1, AMPK α1, Sirt1, Sirt3, ANT1, P300, PGC-1α and other targets in rat myocardial tissues and myocardial cells, and play a crucial role in the treatment of CHF by regulating PPARα/PGC-1α and LKB1/AMPKα/Sirt1 signaling pathway, which promotes mitochondrial energy metabolism and protects against CHF (Wen et al., 2019a; Wen et al., 2020c). In treatment cardiac fibroblast, higenamine inhibits the relative mRNA and protein expression of α-SMA, Acta2, β-MHC, Smad2, and Smad3 in TGF-β1-induced cardiac fibroblast. In addition, higenamine reduces the expression of ECM molecules collagen I and collagen III, thereby improving pathological cardiac fibrosis and dysfunction (Zhu et al., 2021). Higenamine can improve the left ventricular remodeling and contractile function of CRS rats by reducing the expression of TGF-β1, a-SMA, and Col1A1. In addition, higenamine significantly inhibits the relative protein expression of p-ASK1, MAPK, ERK, p38, and NF-kB in the heart tissues of CRS rats and cardiomyocytes. Thus, higenamine improves the heart and kidney function of CRS rats by targeting the ASK1/MAPK (ERK, P38)/NF-kB signaling pathway (Deng et al., 2020). Higenamine inhibits the apoptosis biochemical markers caspase 3 and 9 in primary neonatal rats and adult mouse ventricular myocytes. In intact mouse hearts, higenamine reduces I/R-induced myocardial damage and reduced lytic caspase levels in a β2-AR-dependent manner. Overall, higenamine exerts anti-apoptotic and cardioprotective effects by regulating the β2-AR/PI3K/AKT cascade^[23]^ (Figure 2).

In conlusion, higenamine has a variety of cardiovascular pharmacological activities *in vivo* and *in vitro*, which has positive inotropic and positive heart strengthening effects, as well as relaxing tracheal and vascular functions. It could alleviate the conduction function of sinoatrial node cells by stimulating β-AR in the sinoatrial node to play the role of treating bradyarrhythmia. Higenamine exerts vasodilatory, anti-apoptotic and anti-oxidative stress effects by inhibiting α1-AR and agonizing β2-AR. The vasodilator effect is used for cardiac stress test diagnosis. The anti-apoptotic and anti-oxidative stress effects can reduce myocardial I/R injury. In addition, It is often used in the treatment of HF and bradycardia and served as a cardiac stress test to diagnose coronary artery stenosis and myocardial deficiency.

Higenamine is a drug with important research value and development prospects. There are various of reports about its heart strengthening and anti-HF effects, and it has clear pharmacological effects on the cardiovascular system. At present, it is necessary for researchers to strengthen the research on the pharmacological effects of higenamine and the deep-seated mechanism of action to help its development and clinical application.

## Glossary

CAD: Coronary artery disease
CHF: Chronic heart failure
I/R: Ischemia/reperfusion
CRS: Cardiorenal syndrome
TCM: Traditional Chinese medicine
ALRP: Aconiti lateralis radix praeparata
AR: Adrenergic receptors
HR: Heart rate
CFDA: China food and drug administration
CHD: Coronary heart disease
CNKI: China national knowledge infrastructure
VMIS: VIP medicine information system
CBM: Chinese biomedical database
AUC: Area under the concentration-time curve
CL: Total clearance
NR: Not report
HF: Heart failure
DOX: Doxorubicin
LVSP: Left ventricular systolic pressure
+dp/dtmax: Left ventricular pressure
LVEDP: Left ventricular end-diastolic pressure
Ang-II: Angiotension II
ALD: Aldosterone
ET-1: Endothelin-1
BNP: Brain natriuretic peptide
LDH: Lactate dehydrogenase
CK-MB: Creatine kinase-MB
AST: Aspartate aminotransferase
ATP: Adenosine triphosphate
NAD: Nicotinamide adenine dinucleotide
RAAS: Renin-angiotensin-aldosterone system
MI: Myocardial infarction
HO-1: Heme oxygenase-1
IHD: Ischemic heart disease
ECM: Extracellular matrix
EndMT: Endothelial-to-mesenchymal transition
TAC: Transverse aortic constriction
ISO: Isoproterenol
STNx: Subtotal nephrectomy
i.p: Intraperitoneal injection
FS: Fractional shortening
EF: Ejection fraction
LVVs: Left ventricular volumes
HW/TL: Heart weight to tibia length
HW/BW: Ratio of heart weight to body weight
β-MHC: β-Myosin heavy chain
ANP: A-Type natriuretic peptide
LVEF%: LV ejection fraction
LVFS%: Left ventricular fraction shortening
LVESV: LV end systolic volume
LVPW: LV posterior wall thickness
CWI: Cardiac weight index
KWI: Kidney weight index
Scr: Serum creatinine
BUN: Blood urea nitrogen
IS: Indole sulfate
α-SMA: α-Smooth muscle actin
TGF-β1: Transforming growth factor-β1
p-ASK1: Phosphorylated apoptosis signal-regulated kinase 1
MAPK: Mitogen-activated protein kinases
PE: Phenylephrine
OCR: Oxygen consumption rate
ECAR: Extracellular acidification rate
AMCM: Adult mouse cardiac myocytes
AMVM: Adult mouse ventricular myocytes
NRCF: Neonatal rat cardiac fibroblasts
NRCM: Neonatal rat cardiac myocyte
NRVM: Neonatal rat ventricular myocytes
SK: SK-N-MC cell
SBP: Systolic blood pressure
DBP: Diastolic blood pressure
LD50: Median lethal dose
